# Measles and Scarlet Fever

**Published:** 1880-12

**Authors:** 


### MEASLES AND SCARLET FEVER.

It is important to be able to readily distinguish between these two diseases, which are in some respects similar. Measles presents the appearance of a patchy redness of a circular form, showing white between, with small pimples that feel like little hard points. The mouth and throat are red and inflamed, causing a cough and other symptoms of cold. About the third day the eyes become inflamed and watery. In spite of all remedies the symptoms do not abate. A day or two later the eruption appears upon the neck and head, and then extends over the rest of the body. At last it attacks the bowels, causing diarrhea, and then the other symptoms quickly disappear. The essential point in the treatment is to avoid taking cold. There are few diseases that so readily and completely recover with perfect nursing, and few that entail such a series of misfortunes without it. Medicine is seldom necessary. A portion of the body at a time may be sponged with warm water and then carefully wiped dry before extending the operation. The room should be well aired, but no current of air should touch the patient. The food should be light and easy of digestion.

Scarlet Fever is also red, but it has a smooth feel in the skin, and the redness is suffused like a blush, which deepens till it is very red. There is loss of appetite, pains in the limbs and sore throat; this is the dangerous part. In scarlet fever the rash comes out the second day; in measles the fourth. In scarlet fever there is sore throat, none in measles. In scarlet fever the patient seems to have no cold, as in measles. But little treatment is needed in mild cases. If the urine is not free, drink flax-seed tea or lemonade. Gargle the throat with red pepper, vinegar and water, or a solution of chlorate of potash.

The main thing is to bring out the rash and keep it out. Nothing can compare with frequent warm baths for this purpose; or, if there is much debility, warm sponge baths. Check thirst with lemonade, buttermilk, etc. Keep the room cool and well ventilated. Meat or poultry broth and soups may be given.

Scarlet Fever is also a disease that must run its course, and the patient is to be protected by careful nursing from death, or some of the unfortunate complications that so frequently follow this disease.

